# Role of pyruvate kinase M2-mediated metabolic reprogramming during podocyte differentiation

**DOI:** 10.1038/s41419-020-2481-5

**Published:** 2020-05-11

**Authors:** Qi Yuan, Jiao Miao, Qianqian Yang, Li Fang, Yi Fang, Hao Ding, Yang Zhou, Lei Jiang, Chunsun Dai, Ke Zen, Qi Sun, Junwei Yang

**Affiliations:** 1 Center for Kidney Disease, 2nd Affiliated Hospital, Nanjing Medical University, 262 North Zhongshan Road, Nanjing, Jiangsu 210003 China; 2 Jiangsu Engineering Research Center for MicroRNA Biology and Biotechnology, School of Life Science, Nanjing University, 22 Hankou Road, Nanjing, Jiangsu 210093 China

**Keywords:** Checkpoint signalling, Cell growth

## Abstract

Podocytes, a type of highly specialized epithelial cells, require substantial levels of energy to maintain glomerular integrity and function, but little is known on the regulation of podocytes’ energetics. Lack of metabolic analysis during podocyte development led us to explore the distribution of mitochondrial oxidative phosphorylation and glycolysis, the two major pathways of cell metabolism, in cultured podocytes during in vitro differentiation. Unexpectedly, we observed a stronger glycolytic profile, accompanied by an increased mitochondrial complexity in differentiated podocytes, indicating that mature podocytes boost both glycolysis and mitochondrial metabolism to meet their augmented energy demands. In addition, we found a shift of predominant energy source from anaerobic glycolysis in immature podocyte to oxidative phosphorylation during the differentiation process. Furthermore, we identified a crucial metabolic regulator for podocyte development, pyruvate kinase M2. *Pkm2*-knockdown podocytes showed dramatic reduction of energy metabolism, resulting in defects of cell differentiation. Meanwhile, podocyte-specific *Pkm2*-knockout (KO) mice developed worse albuminuria and podocyte injury after adriamycin treatment. We identified mammalian target of rapamycin (mTOR) as a critical regulator of PKM2 during podocyte development. Pharmacological inhibition of mTOR potently abrogated PKM2 expression and disrupted cell differentiation, indicating the existence of metabolic checkpoint that need to be satisfied in order to allow podocyte differentiation.

## Introduction

Podocyte is a highly differentiated neuron-like epithelial cell with limited capacity for cell division. These cells possess unique, sophisticated foot processes, and slit diaphragm, one of the principal components of glomerular filtration barrier^1–3^. Disruption of podocyte integrity can result in development of proteinuria and glomerulosclerosis^4,5^. It has been reported that induction of podocyte differentiation by retinoic acids (RA) has renal protective effects in several experimental models of kidney disease^6,7^. These studies provide a strong scientific basis supporting that understanding how these cells maintain their differentiated structure and function may be the first step in preventing podocyte loss.

Cellular differentiation involves dynamic epigenetic, transcriptional, and metabolic remodeling as cells transiting^8^. Evidence is mounting that metabolic changes could not only be necessary for differentiation, but may also control it^9–11^. Mitochondrial oxidative phosphorylation (OXPHOS) and glycolysis are two major pathways for cellular energy generation. The activities of these two metabolic pathways are exquisitely controlled to guarantee the optimal resource distribution and cell function. Malfunction of energy metabolism was found participated in the processes of kinds of glomerular diseases, but the pathogenesis studies are still required^12,13^. Therefore, it is imperative to characterize how metabolic profiles of podocytes been set up during differentiation, maintained in adult life, and altered in glomerular diseases. Understanding how specific metabolic processes influence podocyte may offer novel therapeutic approaches in the clinic.

Pyruvate kinase (PK), a rate-limiting glycolytic enzyme, catalyzes the last step of glycolysis by converting phosphoenolpyruvate (PEP) to pyruvate. Mammalian cells contain two PK genes: *Pkrl* and *Pkm*. The *Pkrl* gene encodes the PKL and PKR isoforms, expressed in the liver and red blood cells, respectively. The *Pkm* gene encodes PKM1 and PKM2, expressed in most tissues^14^. Despite that PKM1 and PKM2 are generated by exclusive alternative splicing from one transcript, they have very different catalytic and regulatory properties. PKM1 subunits form stable tetramers and exhibits high constitutive enzymatic activity, whereas PKM2 exists as inactive monomer, less active dimer, and active tetramer. While the PK activity of PKM2 tetramers promotes the flux of glucose-derived carbons via oxidative phosphorylation, the dimeric PKM2 diverts glucose metabolism towards anabolism through aerobic glycolysis^15,16^. The tetramer/dimer ratio of PKM2 are controlled by cellular ATP, fructose-1,6-bisphosphate (FBP) and interactions with signaling proteins^17,18^. The intracellular location of PKM2 can also be exquisitely arranged in order to regulate multiple metabolic pathways^19,20^. Thus, these regulations of expression, allosterism, and translocation of PKM2 allow metabolic flexibility for cells to adapt to different microenvironments, and makes it an excellent regulator of metabolic changes. It has been reported that *Pkm*-knockdown immortalized mouse podocytes had higher levels of toxic glucose metabolites and mitochondrial dysfunction^21^. However, no role has been described for PKM2 in podocyte bioenergetics during differentiation to date. Here, we evaluate the metabolic profiles of podocytes in different developmental stages, and we report an important role of PKM2 in regulating podocyte’s energy metabolism and cell differentiation. In addition, we show that PKM2 expression was regulated by mTOR in podocyte. Pharmacological inhibition of mTOR results in decreased PKM2 level and disrupted podocyte differentiation.

## Results

### Metabolomics analysis revealed higher aerobic glycolysis in differentiated podocytes (DPs)

**Fig. 1:**
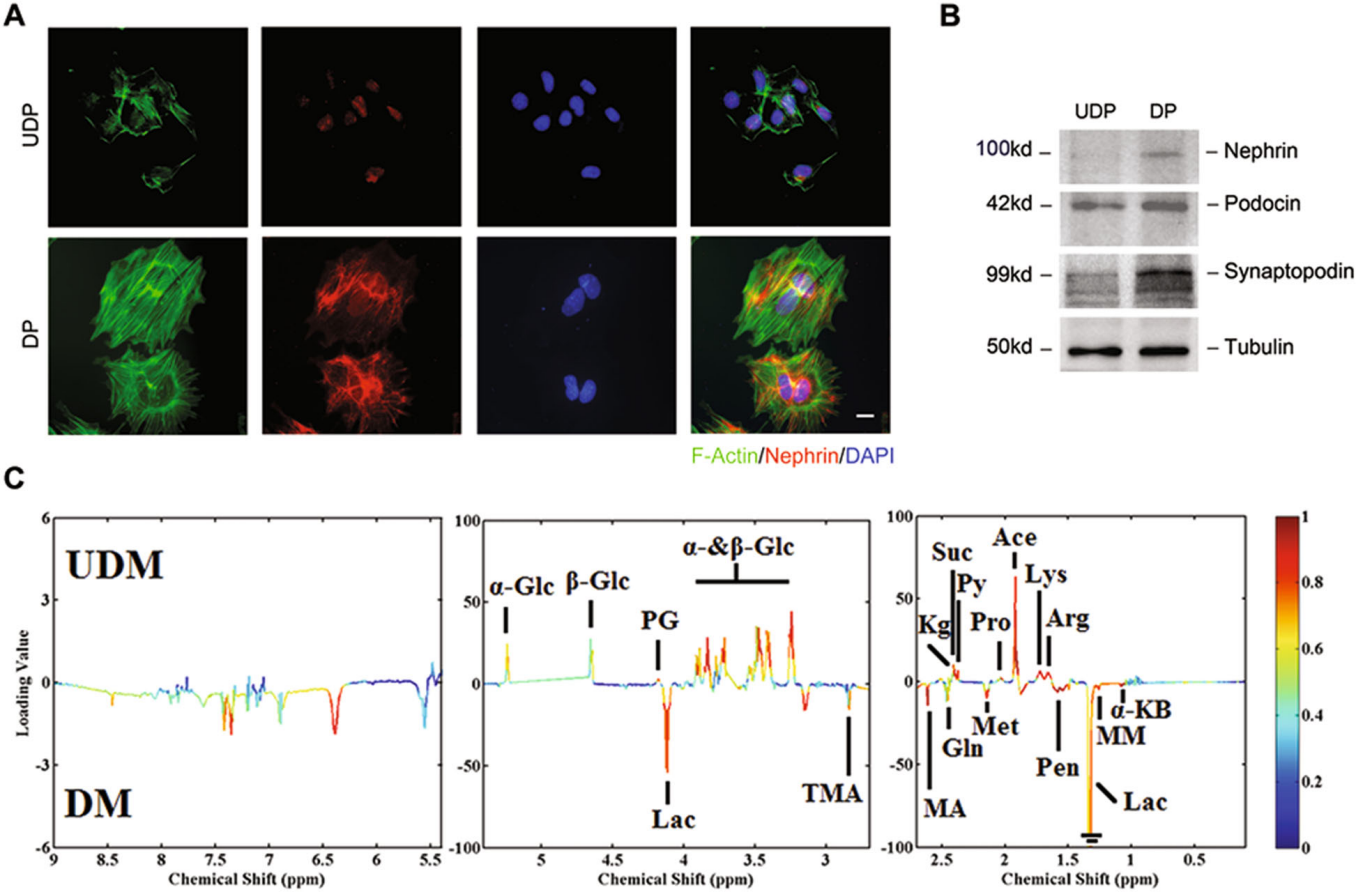
Metabolomics analysis revealed higher lactate production in differentiated podocytes. **a** Immunofluorescence staining for nephrin (red), phalloidine for F-actin (green) and DAPI for nuclear (blue) in undifferentiated podocytes (UDPs) or differentiated podocytes (DPs) as indicated (*n* = 5). Scale bar=5 μm. **b** Representative western blotting results of nephrin, podocin and synaptopodin confirm the differentiation of podocytes (*n* = 3). **c** Representative map of ^1^H-NMR spectra in the extracellular medium incubated with the presence of different podocytes (*n* = 6). UDM: undifferentiated podocyte medium; DM: differentiated podocyte medium. The map shows the significance of metabolites variations between these two classes. Peaks in the positive direction indicate metabolites that are more abundant in the UDM groups. Consequently, metabolites that are more abundant in DM are presented as peaks in the negative direction.

To study the metabolic alterations caused by differentiation, we used a transformed mouse podocyte cell line^22^. Upon the induction of differentiaton for 14 days, the immature podocytes gradually transformed into large, nonproliferating, frequently multinucleated cells. These cells showed many of the specialized characteristics of mature podocytes, including cell flattening, cortical F-actin and interdigitating actin-rich foot processes (Fig. 1a), as well as the expression of specialized proteins associated with slit diaphragm: nephrin, podocin, and synaptopodin (Fig. 1b).

During routine culture, DPs acidified the medium faster than undifferentiated podocytes (UDPs) (data not shown). The changes of extracellular pH are an indicator of altered metabolic pattern. To determine which metabolic pathways were altered in these cells, we performed a metabolomic profiling of the conditional medium of DPs and UDPs by using nuclear magnetic resonance (NMR) spectroscopy (Fig. 1c). The results showed that the metabolomic profile of DPs differed significantly from that of UDPs, with the most prominent alteration being lower glucose concentration and higher lactate production. To determine whether this metabolic increase was proliferation-dependent, we performed Flow Cytometer and found that DPs were growth-arrested (Fig. S1A), suggesting this lactate production was proliferation-independent.

**Fig. 2:**
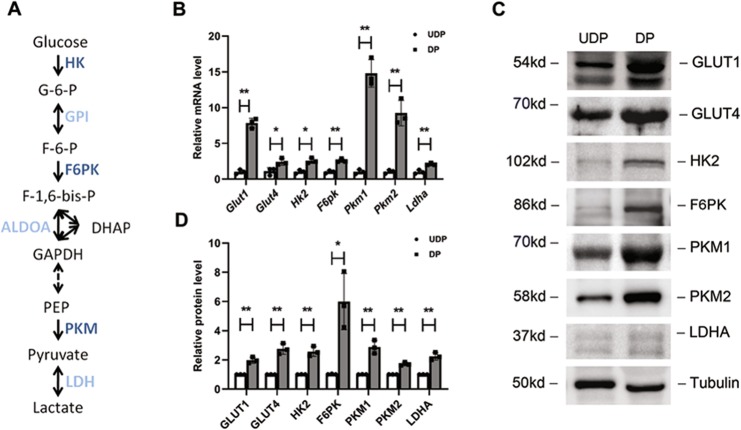
Glycolysis-related genes and proteins were upregulated in differentiated podocytes. **a** Glycolytic pathway with the assayed glycolytic genes in dark blue. **b** Real-time PCR analysis of glycolysis-related mRNAs performed in UDPs and DPs. mRNAs were normalized to actin and compared to UDPs (*n* = 3). **c** Representative blot images of glycolysis-related proteins (*n* = 3). **d** All proteins were normalized to tubulin and compared to UDPs. *P < 0.05, **P < 0.01, determined by *t* test. Data are shown as the means ± SD.

As lactate is the end product of glycolysis^23^, the overproduction of lactate prompted us to examine the glycolytic changes of mature podocytes. The schema in Fig. 2a illustrates the primary enzymes involved in glycolysis. Previous studies demonstrated that transcriptional changes can regulate the glycolytic response in cells^24–26^. In line with this, real-time PCR analysis was performed and revealed that DPs showed a transcriptional increase of glycolytic enzymes (Fig. 2b), and these elevations were further confirmed by immunoblot analysis (Fig. 2c, d). Moreover, we found increased expression at the protein and mRNA level of glucose transporter isoform 1 and 4 (GLUT1/4). As GLUT1 and GLUT4 comprise the major glucose transporters in podocytes^27^, the increased GLUT1/4 expression would be expected to increase glucose uptake. These findings together suggest higher aerobic glycolysis in DPs.

### Podocyte differentiation promoted mitochondrial fusion and biogenesis

**Fig. 3:**
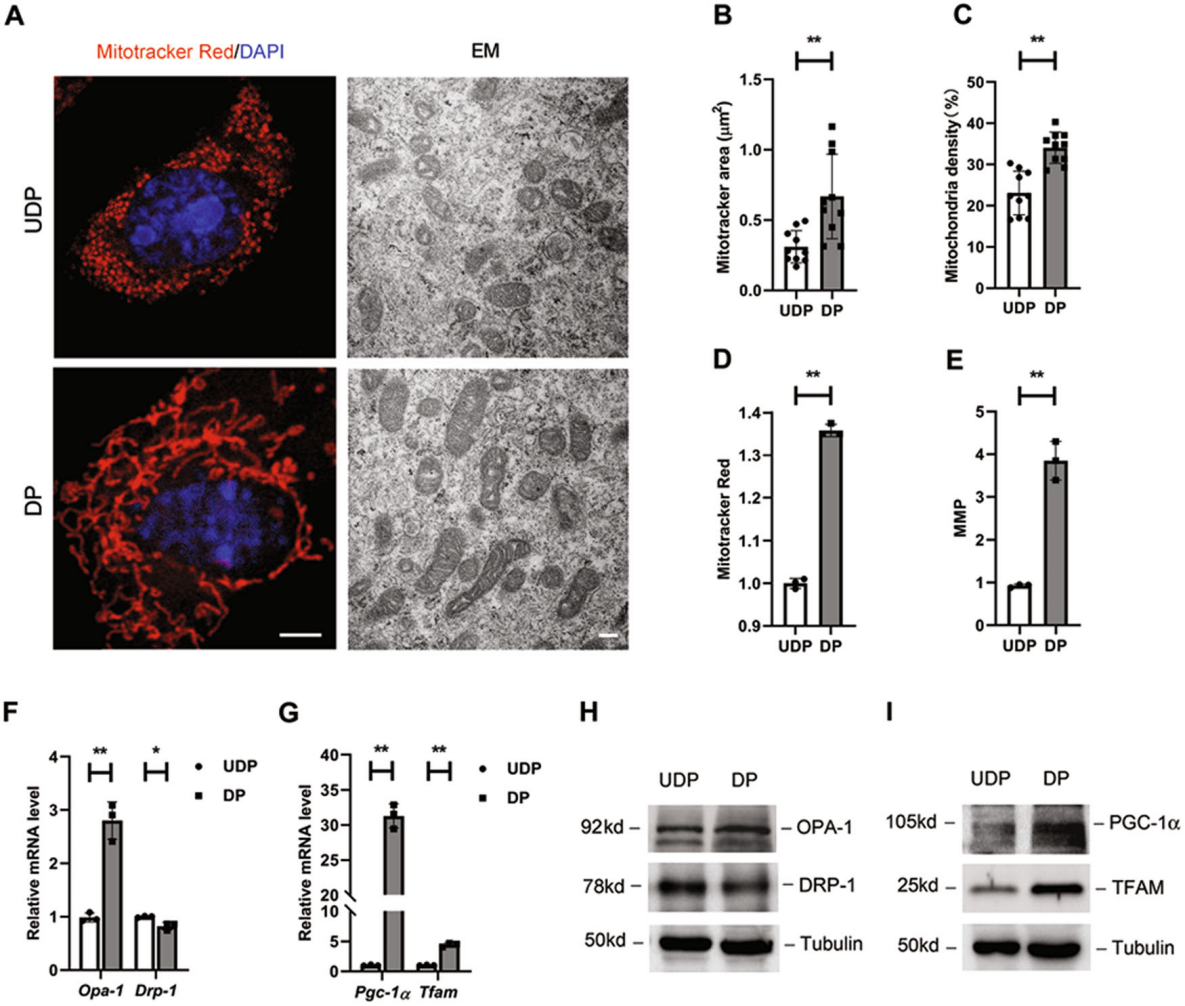
Differentiation of podocytes stimulated mitochondrial function. **a** Representative confocal and electron microscopy (EM) images showing alterations in mitochondrial morphologies between podocytes as indicated. In the confocal images, cells are labeled with MitoTracker Red (red) for mitochondria and DAPI (blue) for nuclear. Left scale bar=2 µm. Right scale bar=500 nm. Pictures show representative fields of over 10 cells photographed. Statistical analyses showing the average size of mitochondria (**b**) and the proportion of total mitochondrial in podocytes (**c**), and data were measured by ImageJ. **d** Mitochondrial mass stained by MitoTracker Red and measured by Flow Cytometer (*n* = 3). **e** Mitochondrial membrane potential labeled with the fluorescent dye JC-1 and measured by Flow Cytometer (*n* = 3). Real-time PCR analysis of mRNAs involved in mitochondrial dynamics (*Opa-1* and *Drp-1*, F) and mitochondrial biogenesis (*Pgc-1α* and *Tfam*, G) in cultured podocytes (*n* = 3). **h, i** Representative western blotting results of relevant protein levels (*n* = 3). *P < 0.05, **P < 0.01, determined by *t* test. Data are shown as the means ± SD.

Cell differentiation was often accompanied by mitochondrial remodeling^28,29^. In order to investigate whether mitochondrial metabolism was associated with podocyte differentiation, mitochondrial morphology was first examined. MitoTracker Red staining and electron microscopy (EM) showed that mitochondria in DPs displayed higher elongation and interconnectivity, indicating a higher energetic potential per mitochondria volume, whereas UDPs had small and round mitochondria (Fig. 3a). In addition, by analyzing EM pictures, the average area and density of mitochondria were both found increased (Fig. 3b, c). In line with the morphology changes, elevations of mitochondrial mass and mitochondrial membrane potential (MMP) were also observed (Fig. 3d, e), suggesting a stronger mitochondrial function.

Then, as the shape of mitochondria dynamically changed, both fusion and fission makers were measured. The transcription level of optic atrophy 1 (*Opa-1*), a mediator of mitochondrial fusion^30^, was increased in mature podocytes, meanwhile, dynamin-related protein-1 (*Drp-1*), an essential protein for mitochondrial fission, was reduced, indicating an improvement in mitochondrial fusion (Fig. 3f). Markers for mitochondrial biogenesis were further investigated. Peroxisomal proliferator-activated receptor coactivator 1α (*Pgc-1α*), a critical transcription factor that regulates mitochondrial biogenesis^31^, presented an increased mRNA level, and the mitochondrial transcription factor A (*Tfam*), a direct regulator of mitochondrial (mt)DNA replication^32^, was upregulated simultaneously (Fig. 3g). Thus, mitochondrial biogenesis is apparent in DPs. Protein levels were further examined by immunoblot analysis, and showed the same trendency with the transcripts (Fig. 3h, i). From all these data, we conclude that differentiation of podocytes promoted mitochondrial fusion and stimulate their biogenesis.

### Differentiated Podocytes preferentially relied on OXPHOS for their energy demands

**Fig. 4:**
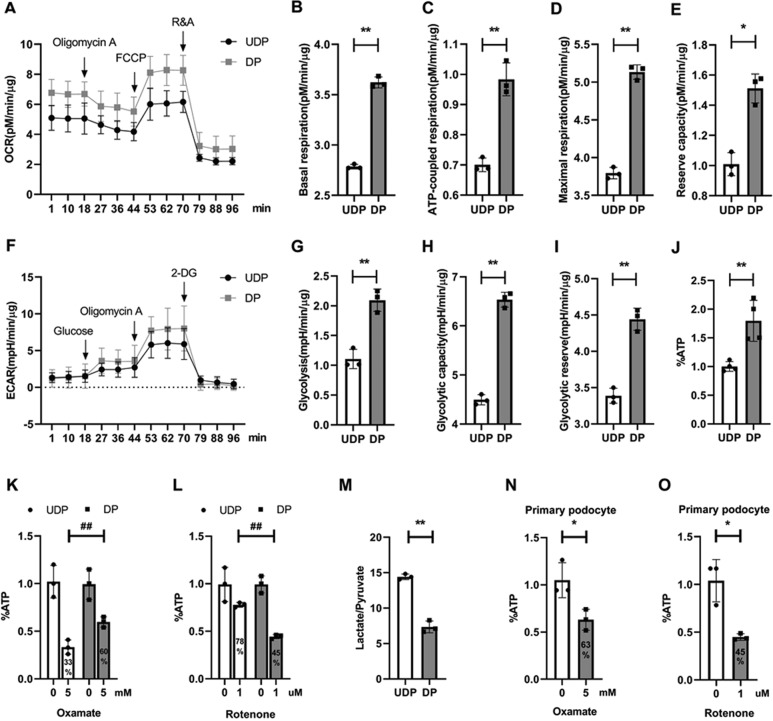
Differentiated podocytes preferentially relied on OXPHOS for their energy demands. **a** Oxygen consumption rate (OCR) of podocytes, followed by sequential treatments with oligomycin, FCCP and rotenone/antimycin A. **b**–**e** Statistical analyses of baseline respiratory capacity, ATP-coupled respiratory capacity, maximum respiratory capacity and reserve respiratory capacity in OCR. **f** Extracellular acidification rate (ECAR) in cultured podocytes, followed by sequential treatments with glucose, oligomycin A, and 2-deoxyglucose (2-DG). **g**–**i** Statistical analyses of glycolysis, glycolytic capacity and glycolytic reserve in ECAR. **j** Intracellular ATP level was normalized by protein content, and undifferentiated podocyte ATP was used as control. **k**–**l** ATP level in cultured podocyte was measured in response to glycolysis inhibitor oxamate or complex I inhibitor rotenone treatment for 45 min, individually. ATP level was normalized by protein content and expressed as % of control, which was defined as the baseline value in cells exposed only to vehicle (*n* = 3). **m** Quantification of lactate and pyruvate ratio in different podocytes (*n* = 3). **n**–**o** ATP levels in primary podocyte was normalized by protein content and expressed as % of control (*n* = 3). *P < 0.05, **P < 0.01, ^#^*P* < 0.05, ^##^*P* < 0.01, determined by *t* test. Data are shown as the means ± SD.

To further define the metabolic profile quantitatively, we examined the oxygen consumption rate (OCR) and extracellular acidification rate (ECAR), respectively. First, we found that the basal respiration of DPs was higher than that of immature podocytes, as shown in Fig. 4b. Then we assessed the function of electron transfer chain (ETC) complexes by sequentially adding pharmacological inhibitors. When oligomycin A was added, the ATP-coupled oxygen consumption was significantly increased after differentiation (Fig. 4c). Next, we determined the maximal OCR that cells can sustain by adding carbonyl cyanide 4-(trifluoromethoxy) phenylhydrazone (FCCP). This treatment resulted in a stimulation of OCR, which was much higher in differentiated cells as shown in Fig. 4d. In addition, reserve respiration capacity, the difference between maximal and basal respiration was also increased (Fig. 4e). Finally, we added Rotenone and Antimycin into podocytes, and no significant differences were observed in non-mitochondrial respiration (Fig. 4a). Taken together, all these results demonstrated an increased OXPHOS activity in DP.

ECAR analysis provided a quantification of glycolytic flux. First, we found that non-glycolytic acidification rate was unchanged during differentiation (Fig. 4f). Nevertheless, the acidification rate was increased higher after glucose and oligomycin A injection in mature podocytes, indicating a significant improvement in glycolysis and maximum glycolytic capacity (Fig. 4g, h). Glycolytic reserve, the difference between glycolytic capacity and glycolysis, was also increased (Fig. 4i). These findings confirmed an increase of glycolysis activity at the differentiation stage.

As both OXPHOS and glycolysis activity were enhanced, these changes translated into higher ATP generation. The intracellular ATP level was upregulated about 80% in mature podocytes, as shown in Fig. 4j. Next, we assessed the contribution of the distinct ATP generating pathways to the overall ATP production in podocytes. Oxamate, a lactate dehydrogenase inhibitor, reduced ATP content by 40% in DPs, while reduced ATP > 65% in UDPs (Fig. 4k), indicating glycolysis inhibition abrogated higher ATP content in immature podocytes. These data suggest that UDPs preferentially rely on aerobic glycolysis for their energy demands. We then treated podocytes with rotenone, and found that rotenone lowered nearly half of the ATP content in DPs, but had only less effect in immature cells (Fig. 4l). Accordingly, the ratio of lactate and pyruvate was also decreased in mature podocytes (Fig. 4m), indicating that less intracellular pyruvate was catalyzed to lactate. These data together suggest that OXPHOS is the primary source of energy in DPs. To gain further insights in the relative contributions of glycolysis and OXPHOS to ATP production under physiological conditions, we isolated primary podocyte from C57BL/6 mice, and treated them with oxamate and rotenone, separately. As Fig. 4n, o shows, similar with transformed cells, rotenone abrogated more ATP content than glycolysis inhibitor in primary podocyte.

So far, these results demonstrate that DPs are capable of reprogramming their metabolism, including facilitating stronger glycolytic profile and increased mitochondrial respiration. In addition, it appears that there is a major switch of the key energy producer from anaerobic glycolysis to mitochondrial oxidative metabolism during differentiation. We first report that glycolytic metabolism, while sufficient for immature podocytes, should be transformed into more efficient OXPHOS to secure the augmented energetic requirements needed by DPs. However, how this energetic switch be programmed was elusive.

### Regulation of PKM2 during in vitro podocyte differentiation

**Fig. 5:**
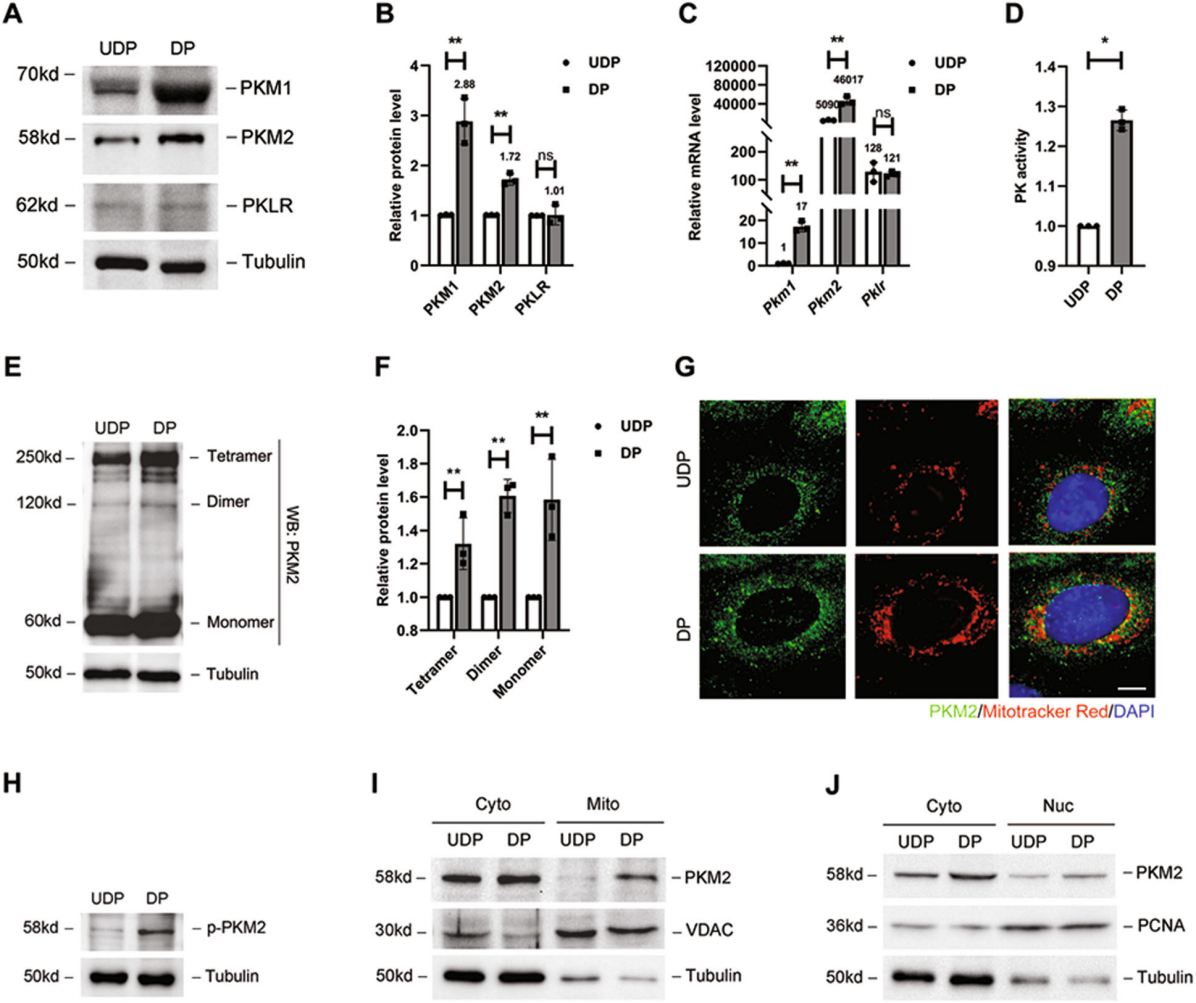
Regulation of PKM2 during in vitro podocyte differentiation. **a** Representative blot image of PKM1, PKM2, PKLR proteins (*n* = 3) in podocytes. **b** Bar charts show means of optical density (O.D.), and normalized to undifferentiated podocytes. **c** Real-time PCR analysis of *Pkm1*, *Pkm2* and *Pklr* mRNAs performed in UDPs and DPs. Relative expression level was compared to *Pkm1* in UDPs (*n* = 3). **d** PK activity in podocytes as indicated (*n* = 3). **e** Representative blot image of cross-linked podocytes (*n* = 3). **f** Tetramer, dimer and monomer PKM2 were compared to tubulin and normalized with undifferentiated podocytes (*n* = 3). **g** Immunofluorescence staining for PKM2 (green), mitochondria (red) and DAPI (blue) in podocytes as indicated. Mitochondria and nuclear from mature podocytes showed more staining of PKM2 (*n* = 10). Scale bar=2 μm. **h** Representative blot image of phosphorylated PKM2 on Tyrosine 105 (*n* = 3). **i**–**j** Mitochondria and nuclear fractions were prepared from undifferentiated or differentiated podocyte. Western blot analysis of cytosolic, mitochondrial and nucleonic fractions was performed to evaluate translocation of PKM2 from the cytosolic compartment to mitochondria and nuclear. *P < 0.05, **P < 0.01, determined by *t* test. Data are shown as the means ± SD.

Among the metabolic enzymes elevated in DPs, PKM2 was of particular interest not only because its transcripts and protein level were upregulated dramatically (Fig. 5a–c), but also because it is a well-studied enzyme that can simultaneously modulate glycolysis flux and mitochondria function^21^. To determine the role of PKM2 in podocyte development, we first determined whether PKM2 is the predominant isozyme. Although the upregulation of PKM1 protein level was greater than PKM2 (Fig. 5b), *Pkm1* transcripts was thousands of times less (Fig. 5c). Thus, we considered that the part PKM1 contributed to total PK content was negligible. We also studied another isozyme, PKLR, and we did not observe any significant change in its mRNA and protein level during differentiation. Taken together, we suggest that PKM2 is a predominant isoform of PK in podocyte.

PKM2 has been shown to play a central regulative role in metabolic reprogramming by multiple pathways, including expression, activity, allosteric regulation, posttranslational modification and translocation^33^. Thus, in addition to changes in PKM2 expression level, PK activity was determined. Consistent with the higher PKM2 expression, PK activity was elevated (Fig. 5d). The mechanism of higher PK activity could be an increase in the distribution of PKM2 tetramer, which is known to be more enzymatically active than PKM2 dimer and monomer. Thus, in order to assess the status of the allosteric regulation of PKM2, DSS cross-linking studies were performed. The results showed that the intracellular level of PKM2 tetramer was higher at differentiation state (Fig. 5e, f).

From the DSS cross-linking studies, an induction of the dimeric and monomeric configuration of PKM2 was also observed during differentiation. As phosphorylation of PKM2 on Tyrosine 105 is an indicative of monomer/dimer formation, concurrent p-PKM2 was measured and showed increase (Fig. 5h). It has been reported that PKM2 monomer/dimer can translocate from cytoplasm to organelles, regulating mitochondrial respiration or glycolysis^20,34–36^. Thus, here we sought to determine whether its overexpression in mature podocyte is coordinated with alterations of translocation. Immunofluorescent analysis was performed to examine the relocation of PKM2, and the result showed that PKM2 was colocated with mitochondria and nuclear in differentiation state (Fig. 5g), which was further supported by subcellular fractionation analysis, showing that more PKM2 was detected in mitochondrial and nuclear fractions (Fig. 5i, j).

Thus, here we provide evidence for PKM2 induction in response to podocyte differentiation. This induction not only enhanced PKM2 expression, activity, but also promoted relocation. But, whether these regulations of PKM2 were related to the increased glycolysis and mitochondrial respiration in DPs was still unknown.

### The effect of *Pkm2* deletion on podocyte differentiation, mitochondria OXPHOS, and glycolysis

**Fig. 6:**
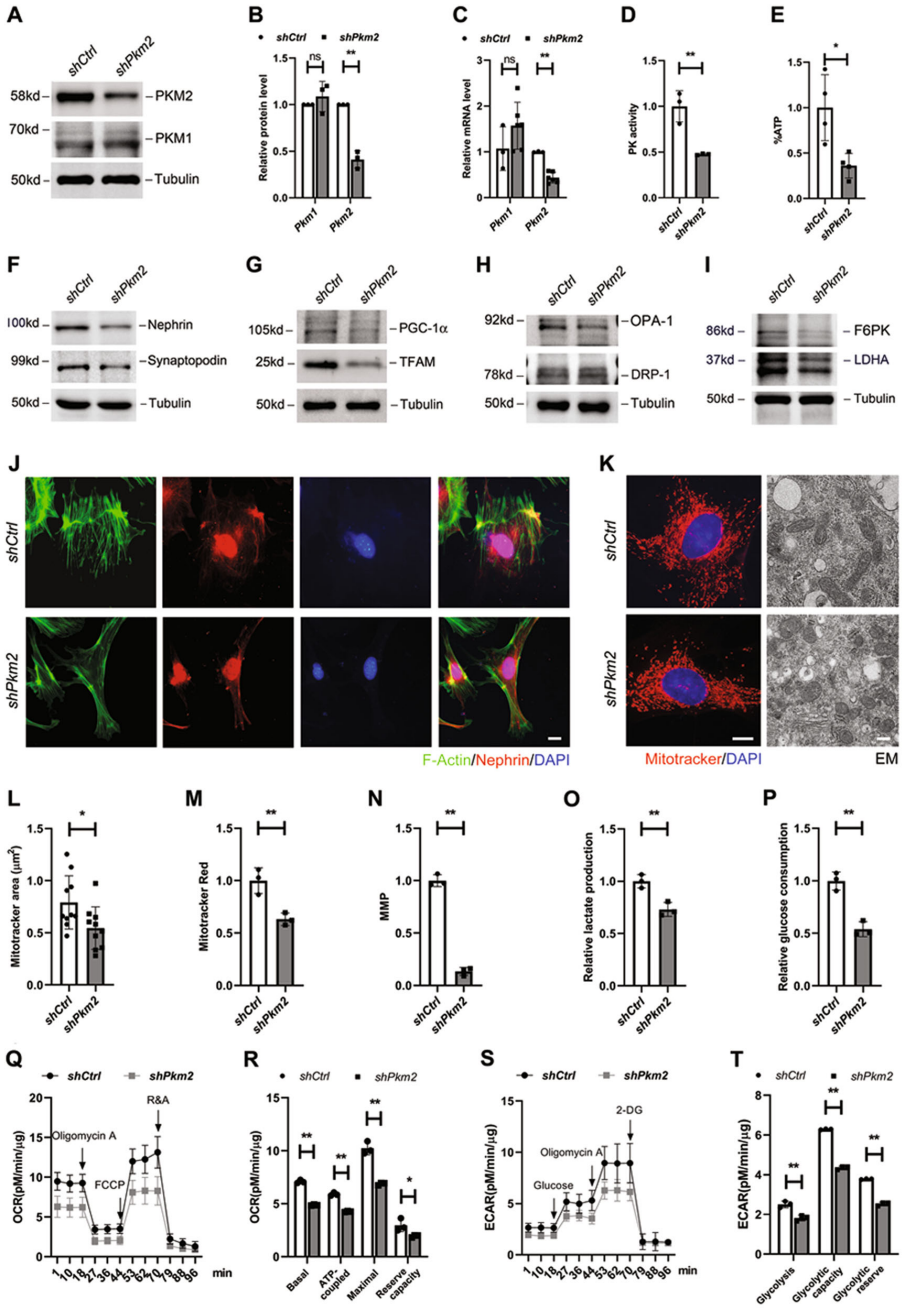
The effect of *Pkm2* depletion on differentiation, mitochondria OXPHOS and glycolysis. **a** Representative western blotting results of PKM2 and PKM1 in control (*shCtrl*) and *Pkm2*-knockdown (*shPkm2*) podocytes (n = 3). **b** Bar charts show means of optical density (O.D.), and normalized to cells transfected with *shCtr*-RNAi lentivirus. **c** Real-time PCR analysis of *Pkm2* and *Pkm1* mRNA levels in the absence or presence of *Pkm2*-RNAi lentivirus (*n* = 3–6). **d** PK activity in different podocytes as indicated (*n* = 3). **e** ATP level was normalized by protein content, and used *shCtrl* podocyte as control. **f** Representative western blotting results of nephrin and synaptopodin expression level in cultured podocytes as indicates (*n* = 3). **g** Representative western blotting results of PGC-1*α* and TFAM expression level (*n* = 3). **h** Representative blot images of OPA-1 and DRP-1 (*n* = 3). **i** Representative blot images of F6PK and LDHA (*n* = 3). **j** Immunofluorescence staining for nephrin (red), phalloidine for F-actin (green) and DAPI for nuclear (blue). Scale bar=5 μm. **k** Representative immunofluorescence and electron microscopy (EM) images showing alterations in mitochondrial morphologies between different podocytes as indicated. In the immunofluorescence images, cells are labeled with MitoTracker Red (red) for mitochondria and DAPI (blue) for nuclear. Left scale bar = 2 µm. Right scale bar = 500 nm. Pictures show representative fields of over 10 cells photographed. **l**–**n** Average mitochondrial area, mitochondrial mass and MMP. **o**, **p** Relative lactate production and glucose consumption in culture medium derived from podocytes, and *shCtrl* podocyte was used as control (*n* = 3). **q** Effects of PKM2 on OCR in podocytes (*n* = 4). OCR traces were obtained using a Seahorse XF96 Analyzer. **r** Statistical analyses of baseline respiratory capacity, ATP-coupled respiratory capacity, maximum respiratory capacity and reserve respiratory capacity in OCR. **s** Effects of PKM2 on ECAR in podocytes (*n* = 4). **t** Statistical analyses of glycolysis, glycolytic capacity and glycolytic reserve in ECAR.

To further determine the effect of PKM2 dysregulation on podocyte differentiation and bioenergetics, we generated a *Pkm2*-RNAi-lentivirus (*shPkm2*) to knockdown PKM2 expression in cultured podocytes. As Fig. 6a shows, the protein level of PKM2 was greatly downregulated after *shPkm2* treatment, while PKM1 was not significantly changed (Fig. 6b). The mRNA level of *Pkm2* were also lower, with a relatively constant expression level of *Pkm1* (Fig. 6c). This result illustrated that there was no compensative expression of PKM1. Notably, with a 60% decrease in PKM2 expression, PK activity was equally reduced (Fig. 6d). In the meantime, the intracellular ATP level was also abrogated about 60% (Fig. 6e), indicating that PKM2 knockdown was associated with PK activity decline and ATP synthesis defect.

Concomitant with decreased ATP, the characteristic markers of mature podocytes, nephrin and synaptopodin, were downregulated simultaneously (Fig. 6f). Immunostaining showed that, although perinuclear distribution of nephrin was most preserved, there was a markedly disrupted pattern at the cell periphery, and phalloidin staining for F-actin showed degeneration of actin filaments bundles within the cytoplasm, indicating that podocyte structure was to some extent disrupted with *shPkm2* treatment (Fig. 6j).

As DPs use OXPHOS as a main energy source, we furthered determined the changes of mitochondria function after *shPkm2* transfection. First, mitochondrial biogenesis and dynamics were examined. The western blotting in Fig. 6g shows that PGC-1α and TFAM, key regulators of biogenesis, were decreased. The downregulation of OPA-1 and the opposite change of DRP-1 indicates the fragmentation of mitochondria (Fig. 6h), which was further judged by MitoTracker Red staining and EM (Fig. 6k). In the meantime, average mitochondria area, mitochondrial mass and MMP were all decreased significantly (Fig. 6l–n).

Next, to investigate the effect of *Pkm2*-knockdown on glycolysis, we examined the expression level of Fructose-6-phosphate Kinase (F6PK) and Lactate Dehydrogenase A (LDHA), two important proteins involved in glycolytic pathway. Their protein levels were both downregulated (Fig. 6i), accompanied by a decrease of relative lactate production (Fig. 6o) and glucose consumption in the culture medium (Fig. 6p). OCR and ECAR were further measured, and the results revealed dramatically downregulation of OXPHOS and glycolysis activity in *Pkm2*-knockdown podocytes (Fig. 6q–t). These data together suggest that PKM2 has a vital effect on ATP production, podocyte differentiation, mitochondrial OXPHOS activity and glycolysis function.

### *Pkm2* deletion in podocytes aggravated adriamycin-induced podocyte injury

**Fig. 7:**
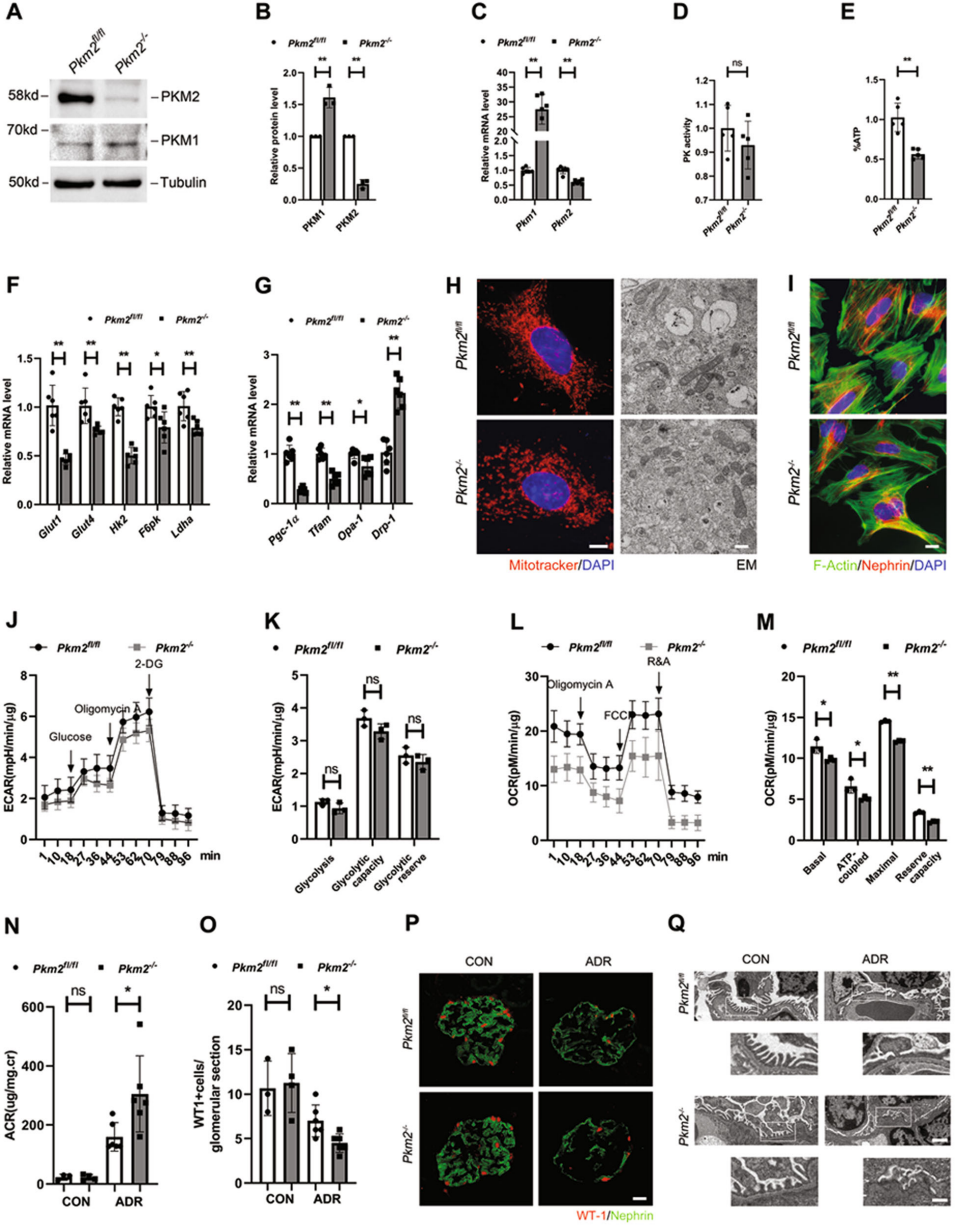
*Pkm*2 deletion in podocytes aggravated adriamycin-induced podocyte injury. **a** Representative western blotting results of PKM2 and PKM1 in primary podocytes isolated from *Pkm2*^*fl/fl*^ and *Pkm2*^*−/−*^ mice (*n* = 3). **b** Bar charts show means of optical density (O.D.), and normalized to *Pkm2*^*fl/fl*^ podocytes. **c** Real-time PCR analysis of *Pkm2* and *Pkm1* mRNA levels (*n* = 5–6). **d** PK activity in different podocytes as indicated (*n* = 5). **e** ATP level was normalized by protein content, and used *Pkm2*^*fl/fl*^ podocyte as control (*n* = 5). **f** Real-time PCR analysis of glycolysis-related mRNAs. mRNAs were normalized to actin, and *Pkm2*^*fl/fl*^ podocyte was used as control (*n* = 6). **g** Real-time PCR analysis of mRNAs involved in mitochondrial biogenesis (*Pgc-1α* and *Tfam*) and mitochondrial dynamics (*Opa-1* and *Drp-1*) in primary podocytes (*n* = 6). **h** Representative immunofluorescence and electron microscopy (EM) images showing alterations in mitochondrial morphologies between different podocytes as indicated. In the immunofluorescence images, cells are labeled with MitoTracker Red (red) for mitochondria and DAPI (blue) for nuclear. Pictures show representative fields of over 10 cells photographed. Left scale bar=2 µm. Right scale bar=500 nm. **i** Immunofluorescence staining for nephrin (red), phalloidine for F-actin (green) and DAPI for nuclear (blue) in primary podocytes as indicated. Scale bar=5 μm. **j** Effects of PKM2 on ECAR in primary podocytes. **k** Statistical analyses of glycolysis, glycolytic capacity and glycolytic reserve in ECAR. **l** Effects of PKM2 on OCR in primary podocytes. **m** Statistical analyses of baseline respiratory capacity, ATP-coupled respiratory capacity, maximum respiratory capacity and reserve respiratory capacity in OCR. **n** Urinary albumin levels in podo-*Pkm2*^*fl/fl*^ and podo-*Pkm2*^*−/−*^ mice after ADR injection (25 mg/kg body weight) at day 5 (*n* = 4–6). **o** Quantitative determination of WT1-positive cells in the glomeruli in different groups as indicated. **p** Immunofluorescence staining for Wilm’s tumor 1 (WT-1) and nephrin in different groups as indicated. Scale bar=20 μm. **q** Representative electron microscopy (EM) shows podocyte foot process effacement after ADR injection. Top scale bar=1 µm. Bottom scale bars =500 nm.

Given the effect of *shPkm2* on cell structure and metabolic reprogramming of cultured podocytes, we sought to determine the functional and metabolic consequences of *Pkm2*-knockout in primary podocytes. To do this, we used primary podocytes generated from mice carrying a *Pkm2* conditional allele with NPHS2-Cre (*Pkm2*^−*/−*^) and matched wild-type controls (*Pkm2*^*fl/fl*^). Western blotting revealed that PKM2 was almost totally eliminated in *Pkm2*^−*/−*^-podocytes (Fig. 7a, b), and the trace amount of PKM2 appeared probably because of the purity (≥95%) of the primary cells (Fig. S2A). Be different from transformed podocytes, *Pkm2*^*−/−*^-podocytes revealed compensatory greater level of PKM1, indicating a switch from PKM2 to PKM1 in primary cells (Fig. 7a–c). Thus, PK activity in the mutant cells were normalized to WT level (Fig. 7d), in keeping with previous characterization of *Pkm2*-KO podocyte^21^. But despite the compensatory expression of PKM1, total intracellular ATP were decreased (Fig. 7e). The mRNA levels of proteins involved in glycolysis (Fig. 7f), mitochondria biogenesis and fusion (Fig. 7g) were reduced as well. MitoTracker Red staining and EM revealed mitochondrial fragmentation (Fig. 7h). These results suggest that the compensation of PKM1 protein or PK activity could not make up for PKM2 on the regulation of podocyte bioenergetics, and that PKM2 might has a more potent and unique glycolytic and mitochondrial effect than PKM1.

However, beyond our expectation, despite the lower energy supply, the morphology of primary *Pkm2*^*−/−*^ podocytes was not changed much, including the distribution of nephrin and the arrangement of F-Actin (Fig. 7i). These results demonstrate that compensatory PKM1 seems to be enough for podocyte maturation and maintaining glycolysis activity (Fig. 7j, k), under normal physiologic conditions. Nevertheless, knockout of PKM2 dramatically decreased the capacity of mitochondrial respiration (Fig. 7l, m). In order to sustain the complex cellular morphology of interdigitating foot processes, podocytes usually rely on a constant energy supply and reservoir^37^. The reduced OXPHOS capacity suggests that these cells might be more vulnerable to extracellular stimuli.

To confirm our hypothesis, we challenged podo-*Pkm2*^*−/−*^ mice with adriamycin (ADR), an agent that specifically damages glomerular podocytes. As shown in Fig. 7n, compared with the control littermates, podo-*Pkm2*^*−/−*^ mice developed more severe albuminuria at day 5, after intravenous injection of ADR at a dose of 25 mg/kg body weight. We further examined the expression of WT-1, a molecular signature of podocytes. ADR caused a significant decrease in the number of the WT-1-positive cells in podo-*Pkm2*^*fl/fl*^ mice. However, Ablation of *Pkm2*, markedly exacerbated WT-1 loss (Fig. 7o). Immunofluorescent staining revealed that levels of nephrin were reduced and its distribution was changed from a linear to granular pattern after ADR injury, and these lesions were more severe in mutant mice (Fig. 7p). We also examined the ultrastructure of podocyte foot processes and slit diaphragm by EM. As shown in Fig. 7q, more severe lesions of foot process and slit diaphragm were observed in podo-*Pkm2*^*−/−*^ mice, suggesting that PKM2 has an important role in podocyte survival after injury.

### mTOR signaling pathway regulated PKM2 expression and podocyte differentiation

**Fig. 8:**
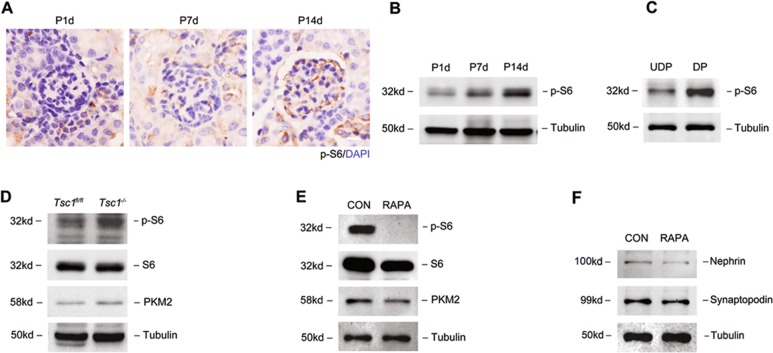
mTOR signaling pathway regulated PKM2 expression and podocyte differentiation. **a** IHC detection of p-S6 in renal cortex during postnatal development. Scale bar=20 μm. **b** Representative western immunoblot analysis of p-S6 in renal cortex. **c** Representative western immunoblot analysis of p-S6 in cultured podocyte during differentiation. **d** Western immunoblot analysis of p-S6 and PKM2 expression in *Tsc1*^*fl/fl*^ and *Tsc1*^*−/−*^-podocytes. **e** Rapamycin caused a significant reduction in the protein levels of p-S6 and PKM2 in cultured podocyte. **f** Western immunoblot analysis of nephrin and synaptopodin with 10 nM rapamycin treatment for 14 days.

It has been reported that podocyte-specific constituent loss of mTORC1 (*Raptor*^*−/−*^) developed significant albuminuria at 4 weeks of age^38^. However, ablation of podocyte Raptor in already matured glomeruli results in only a mild phenotype^39^. These findings suggest that mTORC1 activity is particularly important in developing podocytes. Moreover, mTOR pathway is a critical regulator of cellular metabolism in kinds of tissues^40,41^. Therefore, we asked whether mTOR participates in the metabolic changes observed during podocyte differentiation. To test this hypothesis, we first examined the phosphorylation levels of S6, a downstream target of mTORC1 that is frequently used as an indicator for mTORC1 activity, in podocyte during different differentiation stages in vivo. The results in Fig. 8a show that p-S6 was hardly detected in podocyte at postnatal day 1, but was clearly detected at postnatal day 14. Western blotting also showed increased p-S6 expression during postnatal development (Fig. 8b). To confirm this increase in podocyte in vitro, we assayed the protein level of p-S6, and found that p-S6 was increased in mature podocytes (Fig. 8c). Thus, we demonstrated that mTORC1 activity was progressively and significantly increased during podocyte differentiation.

To address whether activation of mTORC1 in podocytes contributes to PKM2 expression, we generated a podocyte-specific *Tsc1*-KO mice. Because TSC1 is an upstream negative regulator of mTORC1, loss of TSC1 should result in activation of the mTORC1 pathway^42^. *Tsc1*^*−/−*^-podocytes showed increased p-S6 and PKM2 expression than *Tsc1*^*fl/fl*^ cells (Fig. 8d). In contrast, pharmaceutical inhibition of mTORC1 with rapamycin downregulated p-S6 and PKM2 level simultaneously (Fig. 8e). These data suggest mTOR as a positive regulator of PKM2 expression. Furthermore, treatment of podocytes with rapamycin resulted in a significant downregulation of nephrin and synaptopodin in podocyte (Fig. 8f). All these data together suggest that induction of PKM2 during differentiation, was at least partially activated by mTORC1 pathway. Moreover, suppressing mTOR pathway with rapamycin can inhibit PKM2 expression and disrupt podocyte differentiation.

## Discussion

Differences in energy status from distinct physiologies can distinguish differentiated cells from undifferentiated ones^43^. Thus, metabolism could provide a relatively unexplored distinguishing feature between mature and immature cells. Conversely, metabolic studies on podocytes have mainly been conducted on mature podocyte in physiological or pathological conditions. In 2010, Abe^44^ provided first experimental evidence for a critical role of mitochondria in podocyte. Ozawa^45^ suggested that both glycolytic and OXPHOS pathways contribute to podocyte energy supplements. Recently, Paul^37^ highlights the podocytes’ independence from mitochondrial energy sources, but no one have been focused on changes of podocyte’s metabolic profile during differentiation. Here we report for the first time a global metabolic profile in cultured podocyte during in vitro differentiation.

Although the model of in vitro terminal differentiation of cultured podocyte has some limitations, we have chosen it to avoid any contribution from other glomerular cells to the metabolic profile of podocyte. Glycolysis and oxidative phosphorylation (OXPHOS) are the two major cellular pathways to produce energy. Most cells may switch between these pathways in order to cope with changing energy demands. After 14 days differentiation, we observed an activation of glycolysis in cultured podocyte. Usually, differentiated cells have repressed glycolysis, as they mostly rely on OXPHOS for energy demand^43,46^. However, podocytes showed that their in vitro differentiation were associated with increased glycolysis owing, at least in part, to increased expression of GLUT1/4 and of enzymes in the glycolytic pathway. It is known that glycolysis can provide intermediate metabolites for amino acids, lipids and nucleic acids biosynthesis^47^. Thus, mature podocytes might require a higher glycolytic flux probably for synthesizing slit diaphragm proteins and GBM components, which are important for maintaining their phenotype.

As the distribution, abundance and fusion–fission status of mitochondria regulates bioenergetics^48–50^, the morphology and abundance of mitochondria were assessed for changes with differentiation. Mitotracker Red staining has made it clear that DPs have rich mitochondria to support their higher demand for energy. Mitochondria from UDPs were structurally undeveloped, consisting of small and spherical structures. However, as podocyte differentiated, a more filamentous and branched mitochondrial network was formed, with an increased capacity to produce ATP from OXPHOS^51,52^. Thus, these results here collectively show that DPs can boost both glycolysis and mitochondrial metabolism to meet their augmented energy demand. However, the regulation of these differences in bioenergetics is still unknown.

It has been reported that PKM2 can regulate energy metabolism of both mitochondria^21^ and glycolysis^53^. In this study, we demonstrated that PKM2 expression was increased with podocyte differentiation. In order to delineate the role of PKM2 in podocyte, we knocked down PKM2 in podocytes via transfection with a *Pkm2*-RNAi-Lentivirus (*shPkm2*), and found out that *shPkm2* prevented podocyte differentiation, as manifested by nephrin and synaptopodin suppression, and led to glycolysis decrease and mitochondrial dysfunction, based on the reduction of ECAR and OCR. The data thus far showed that *shPkm2* prevented podocye differentiation, at least partially by ruining glycolysis and mitochondrial function. However, in striking contrast, it has been reported that PKM2-Oct-4 can stimulate the undifferentiated pluripotent state of embryonic stem cells^54^. Thus, we suggest that the role of PKM2 on cell differentiation may depend on cell types and context.

Although our results showed vital function of PKM2 in cultured podocyte differentiation, it has been reported that *Pkm2*-null and podocyte-specific *Pkm2*-KO mice are both viable and fertile^21,55^. This phenomenon might be due to the potential compensatory effects of the conventional genetic ablation approaches^56^. In order to figure out the difference between transformed and primary podocyte, we next extended these findings to primary *Pkm2*^*−/−*^-podocytes. In fact, the compensatory expression of *Pkm1* in *Pkm2*^*−/−*^-podocytes seems compatible for podocyte maturation under normal physiologic conditions. Nevertheless, knockout of PKM2 developed more severe albuminuria and aggravated adriamycin-induced podocyte injury dramatically, suggesting that PKM2 has an important role in podocyte survival after injury. Thus, in our experimental conditions, the compensatory increased transcript levels of *Pkm1* weren’t sufficient for *Pkm2*^*−/−*^ podocytes to resist stress under pathological condition. Notably, podocyte-specific *Pkm2*-KO mice with diabetes also developed worse albuminuria and glomerular pathology^21^. The defects of the traditional genetic ablation approach probably make researchers ignore the importance of PKM2 during differentiation. However, the CRISPR gene-editing or Tet-On mice are needed to further proved this hypothesis.

In the present study, we first described the metabolic reprogramming of cultured mouse podocytes, including a strong glycolytic profile and increased mitochondrial metabolism, during in vitro differentiation, and we demonstrated the vital role of PKM2 in this progress. Our findings further support the role of mTOR pathway in the regulation of PKM2 expression and podocyte differentiation. In addition, our work has implications not only for the fundamental understanding of podocyte development, but also for the importance of PKM2-mediated metabolic defects in in vivo podocyte loss.

## Materials and methods

### Animal models

Animal studies were conducted in strict accordance with the principles and procedures approved by the Committee on the Ethics of Animal Experiments of Nanjing Medical University. *Pkm2*^*fl/fl*^ (stock #024048), *Tsc1*^*fl/fl*^ (stock # 005680) and podocin-Cre (stock #008205) mice were purchased from the Jackson Laboratory. All mice were backcrossed to a C57BL/6 background. For podocyte-specific *Pkm2*-KO mice, we cross bred *Pkm2*^*fl/fl*^ mouse strain with the podocin-Cre mice. For podocyte-specific *Tsc1*-KO mice, we cross bred *Tsc1*^*fl/fl*^ mouse strain with podocin-Cre mice. All experiments were performed with genetically appropriate littermate controls. In ADR-induced proteinuria mouse studies, male mice at 8 weeks old received a single, slow tail vein injection of either saline or 25 mg/kg ADR (Doxorubicin hydrochloride, D1515, Sigma-Aldrich, USA). Mice were sacrificed and kidneys were harvested for the analysis at day 5.

### Cell culture and treatment

Conditionally immortalized mouse podocyte cell lines was kindly provided by Dr Zhihong Liu (Nanjing University, Nanjing, China). UDPs were seeded at 5.0×10^4^ cells per well in 6-well plates and cultured at 33 °C in RPMI-1640 medium with 10% fetal bovine serum (FBS, Gibco) and 10 U/ml recombinant mouse γ-interferon (γ-IFN, R&D Systems, MN, USA). To induce differentiation, cells were transferred to non-permissive conditions at 37 °C and incubated with 1 μM RA in the absence of γ-IFN for 14 days (replace the growth medium every 3 days). Lentivirus of Pkm2-RNAi (*shPkm2*) were perchased from Genechem (Shanghai, China) and the sequence was: 5′-GGCCATTATCGTGCTCACCAA-3’. For transfection, cells were infected with lentivirus for 12 h according to the manufacturer’s instructions, and then the medium were replaced with complete medium for indicated time.

### Primary culture of podocytes

This procedure was adapted from a standard operating protocol by Azeloglu Lab. The isolated glomeruli were plated on collagen-type-I-coated dishes at 37 °C in RPMI 1640 medium with 10% FBS. Do not touch for minimum of 3–4 days to allow glomeruli to settle with gravity and stick to the collagen-coated surface and for podocytes to move onto the culture plate. On day 4 of culture, unattached glomeruli were washed away. Podocytes were used for experiments on day 6–8 of culture.

### Urinary albumin

Urine albumin level was measured by using a mouse Albumin ELISA Quantification kit, according to the manufacturer’s protocol (Bethyl Laboratories, Montgomery, TX, USA).

### Transmission electron microscopy

EM of kidney samples was carried out by routine procedures as described previously. Briefly, mouse kidneys were perfusion fixed with 2.5% glutaraldehyde in phosphate buffered saline and post-fixed in aqueous 1% O_S_O_4_. Specimens were dehydrated in increasing concentrations of alcohol, infiltrated in a 1:1 mixture of propylene oxide/Polybed 812 epoxy resin, and then embedded. Sections (100 nm thick) were cut using a Leica (Solms, Germany) UC6 ultramicrotome and stained with 2% uranyl acetate for 10 min, followed by 1% lead citrate for 5 min at room temperature. Sections were then observed and photographed using a FEI Tecnai T20 transmission electron microscope, operated at 120 kV.

### Cross-linking of PKM2

In cell culture, we used 500 µM DSS (Disuccinimidyl suberate, 21655, Thermo Fisher Scientific) to cross-link for 30 min at room temperature. Lysates were analyzed by western blot. After transfer, membranes were incubated with 0.4% paraformaldehyde in PBS for 30 min at room temperature before PKM2 antibody was added for detection of tetramers, dimers and monomers of PKM2.

### PK activity assays

We measured PK activity by using a PK activity assay kit (K709, BioVision, USA). In brief, PEP and ADP were catalyzed by PK to generate pyruvate and ATP. The generated pyruvate is oxidized by pyruvate oxidase to produce color (at λ = 570 nm) and fluorescence (at Ex/Em = 535/587 nm). The PK activity was accurately measured by a microplate reader.

### Mitochondrial morphology

To observe mitochondrial network morphology, MitoTracker Red CMXRos 100 nM (Cat. no. M7512, Invitrogen) was added to culture medium and cells were incubated at 37 °C for 30 min. After that, cells were gently washed, fixed, and double-stained with DAPI to visualize the nuclei. Then, slides were imaged under the confocal microscope (CarlZeiss LSM710).

### Mitochondrial membrane potential

MMP was assessed by measuring the potential-dependent accumulation of 5,5′,6,6′-tetrachloro-1,1′,3,3′-tetraethylbenzimdazol carbocyanine iodide (JC-1, C2005, Beyotime, China). Podocytes were washed twice with HBSS (Sigma-Aldrich, USA) and then incubated in the dark with JC-1 for 30 min at 37 °C. Fluorescence in podocytes was detected with FACS.

### Measurements of OCR and ECAR

A Seahorse Bioscience XF24-3 Extracellular Flux Analyzer was used to measure the rate change of dissolved O_2_ and pH in medium immediately surrounding adherent cells cultured in a XF24-well cell culture microplate (Seahorse Bioscience, North Billerica, MA, USA). Cells were seeded in XF24-well microplates at 2.0×10^4^ cells per well in 200 μl of growth medium, then growth medium was replaced with assay medium.

For OCR, the analyzer plotted the value as the cells were treated by sequential injection of the following compounds: oligomycin (1 μmol/L), carbonyl cyanide-4 (trifluoromethoxy) phenylhydrazone (FCCP, 1.5 μmol/L), and antimycin A (1 μmol/L) plus rotenone (1 μmol/L). For ECAR, the analyzer plotted the value as the cells were treated by sequential injection of the following compounds: glucose (10 mmol/L), oligomycin (2 μmol/L) and 2-deoxy-glucose (2-DG, 100 mmol/L). The results were automatically calculated, recorded, and plotted by Seahorse XF24 software version 1.8 (Seahorse Bioscience). Data were normalized for protein concentration per well.

### ATP quantification

Podocytes were seeded in 12-well microplates (Corning, NY, USA) at 2.0×10^4^ cells per well. Cells were then exposed to vehicle or compounds for 45 min before the ATP assay started. The quantity of ATP present in the test cells in each well was measured by ATP bioluminescent somatic cell assay kit (Cat. no. FLASC, Sigma-Aldrich, St. Louis, MO, USA). The ATP assay was performed according to the manufacturer’s instruction. Luminescence intensity from each well was measured with a Glomax Luminometer (Promega). Data were normalized for protein concentration per well.

### Western blotting

Cells lysed with RIPA buffer were separated by sodium dodecyl sulfate polyacrylamide gel electrophoresis, and electrotransferred onto polyvinylidene fluoride membranes. After overnight incubation with primary antibody at 4 °C, the membranes were incubated with HRP-conjugated anti-mouse or anti-rabbit (Sigma-Aldrich, St. Louis, MO, USA) secondary antibody for 1 h at room temperature, followed by addition of ECL prime (Vazyme, Nanjing, China) to detect bands using a Bio-Rad gel documentation system (Bio-Rad, CA, USA). Primary antibodies were detected against Nephrin (ab80298, Abcam), Podocin (P0372, Sigma), Synaptopodin (NBP2-39100, Novus), GLUT1 (ab115730, Abcam), GLUT4 (NBP1-49533, Novus), HK2 (NBP2-02272, Novus), F6PK (ab154804, Abcam), PKM1 (7067 S, CST), p-PKM2 (3827 S, CST), PKM2 (4053 S, CST), PKLR (ab137787, Abcam), LDHA (3558 S, CST), PGC-1α (ab54481, Abcam), TFAM (ab131607, Abcam), OPA-1 (ab42364, Abcam), DRP-1 (ab184247, abcam), VDAC (4866 s, cst), PCNA (ab18197, abcam), p-S6 (4858 s, cst), S6 (2217 s, cst), and Tubulin (T9026, Sigma) at a dilution of 1:1000. Quantification was performed by measurement of the intensity of the signals with the use of ImageJ (NIH, Bethesda, MD, USA).

### Lactate, Pyruvate assay

Lactate concentration of cell extracts was measured using Lactate Colorimetric/Fluorometric Assay Kit (K607-100, Biovision, USA). Pyruvate concentration of cell extracts was measured using Pyruvate Colorimetric/Fluorometric Assay Kit (K609-100; Biovision, USA).

### Quantitative RT-PCR analysis

Total RNA was extracted using TRIzol reagent (Invitrogen) according to the manufacturer’s instructions. cDNA was synthesized with 1 μg of total RNA. Gene expression was measured by a real-time PCR assay (Vazyme, Nanjing, China) and 7300 Real-Time PCR System (Applied Biosystems, CA, USA). The relative amount of mRNA to internal control was calculated using the expression 2△CT, in which △CT = CT_gene_ - CT_control_, and CT is cycle threshold.

### Immunofluorescent staining

Cells cultured on coverslips were washed twice with cold PBS and fixed with cold methanol:acetone (1:1) for 10 min at −20 ˚C. Following three extensive washings with PBS, slides were blocked with 0.1% Triton X-100 and 2% normal donkey serum in PBS buffer for 40 min at room temperature and then incubated with the specific primary antibodies previously described, followed by staining with isothiocyanate-conjugated secondary antibody. Slides were triple-stained with DAPI to visualize the nuclei. Paraffin-embedded mouse kidney sections (3 μm thickness) were prepared similarly. At the end of the process, slides were viewed with a Nikon Eclipse 80i microscope equipped with a digital camera (DS-Ri1, Nikon). In each experimental setting, immunofluorescence images were captured with identical exposure settings.

### Nuclear magnetic resonance

^1^H-NMR spectra of cell culture media were acquired on a 600-MHz NMR Spectrometer (Bruker Avance III, Bruker Corporation, Kalsruhe, Germany), using a CPBBO detection probe. Acquisition parameters included a 1.70-s acquisition time and an interpulse delay of 3-s to ensure complete relaxation of all nuclei in the sample. The results were analyzed by MestReNova software (V9.0.1, Mestrelab Research, Santiago de Compostela, Galicia, Spain).

### Lactate production and glucose consumption

The conditional media was centrifuged for 5 min at 3000×*g* to remove debris and measured for glucose and lactate content. Lactate concentration of cell supernatant was measured by using Lactate Colorimetric/Fluorometric Assay Kit (K607-100, Biovision). Glucose concentration of cell supernatant was measured by using Glucose Colorimetric/Fluorometric Assay Kit (K606-100, Biovision). Reactions with media samples without incubating with cells were included as a negative control.

### Statistical analysis

Animals were randomly assigned to control and treatment groups. All images of Western blots and immunofluorescence were representatives of at least three independent experiments. RT-qPCR assays were performed in triplicates. Data shown are the mean ± SD for three or more independent experiments. Differences were considered statistically significant at *p < 0.05 or **p < 0.01, assessed using Student’s *t* test.

## Supplementary information

supplemental figure 1

supplemental figure 2

supplemental figure legend
