# Supplementary material for: Role of pyruvate kinase M2-mediated metabolic reprogramming during podocyte differentiation
Source: Cell Death Dis. 2020 May 11;11(5):355. doi: 10.1038/s41419-020-2481-5 (PMC7214446; doi:10.1038/s41419-020-2481-5)
Supplement: Supplementary file 3 — supplemental figure legend [file 41419_2020_2481_MOESM3_ESM.docx]

**Supplemental Figure Legend**

**S.1.** **Differentiated podocytes were growth-arrested.**

(A) Representative set of cell cycle histograms show cell cycle arrest in G1 phase in differentiated podocyte (n=3).

**S.2. Immunofluorescence staining for the purity of primary podocytes.**

(A) Immunofluorescence staining WT-1 (red) for podocyte and DAPI for nuclear (blue) in primary podocyte. Scale bar=20 μm.
